# Tribute to Professor Raphael Mechoulam, The Founder of Cannabinoid and Endocannabinoid Research

**DOI:** 10.3390/molecules27010323

**Published:** 2022-01-05

**Authors:** Mauro Maccarrone

**Affiliations:** 1 Department of Biotechnological and Applied Clinical Sciences, University of L’Aquila, 67100 L’Aquila, Italy; mauro.maccarrone@univaq.it; 2 European Center for Brain Research, Santa Lucia Foundation IRCCS, 00143 Rome, Italy

During the last 60 years the relevance for human health and disease of cannabis (*Cannabis sativa* or *Cannabis indica*) ingredients, like the psychoactive compound Δ^9^-tetrahydrocannabinol (THC), cannabidiol, 120+ cannabinoids and 440+ non-cannabinoid compounds, has become apparent [1]. THC was identified in 1964, and approximately 30 years later (in 1992), the molecular reasons for the biological activity of cannabis extracts were made clearer by the discovery of anandamide (*N*-arachidonoylethanolamine). The latter is the first member of a new family of bioactive lipids collectively termed “endocannabinoids”, that are able to bind to the same receptors activated by THC. In addition to endocannabinoids (that include several *N*-acylethanolamines and acylesters), a complex array of receptors, metabolic enzymes, transporters (transmembrane, intracellular and extracellular carriers) were discovered, and altogether they form a so-called “endocannabinoid system” that finely tunes the manifold biological activities of endocannabinoids themselves [2].

Both plant-derived THC and the first endocannabinoids were discovered in Israel by the laboratory led by Professor Raphael Mechoulam, who has just celebrated his 90th birthday and clearly stood out as a giant of modern science.

I met Professor Mechoulam (Raphi) back in 1999, when I attended my first International Cannabinoid Research Society (ICRS) meeting in Acapulco (Mexico) as a newcomer in the field. Although already acclaimed as the founder of a new research area, Raphi was extremely friendly to me, and curious about the implications of my data on the anandamide-degrading fatty acid amide hydrolase in the wider context of human heath. After quite some years, I can say that Raphi still represents an inspiration for young scientists, and a solid reference for more experienced colleagues who are interested in any aspect of cannabinoid and endocannabinoid research. It is indeed rather difficult to summarize the many seminal discoveries and the huge impact that Raphi has had over the last 60 years, in particular on advancing therapeutic drug discovery. Just to give a few examples, he was the first to elucidate in 1964 the complete structure of THC [3]. Then, he identified many additional phytocannabinoids in 1965–1972 (reviewed in ref. [1]), and later on discovered also the endogenous counterparts of THC: anandamide in 1992 [4], and 2-arachidonoylglycerol in 1995 [5], the latter at the same time and independently of Sugiura and colleagues in Japan [6]. Then, Raphi identified arachidonoyl-serine, an endogenous vasodilator, in 2006, and oleoyl-serine, an endogenous regulator of bone mass, in 2010 (reviewed in refs [7,8]). These are just some of the milestones in Raphi’s (endo)cannabinoid investigations that have boosted intense research on the proteins that bind to and metaboilze these substances, leading to the definition of an entirely new signal transduction system based on bioactive lipids. Such a system, along with plant-derived cannabinoids themselves, is now widely recognized for its therapeutic potential in almost all human diseases, as suggested also by the ever-growing number of investigations that can be retrieved from a PubMed search (Table 1).

The many implications of the seminal work of Raphi for chemistry, biochemistry, biology, pharmacology and medicine are reflected in this special issue by contributions made by Raphi himself and by the selected group of scientists who over the last 20 years received from the ICRS the highest recognition in the field of (endo)cannabinoid research: the Mechoulam Award.

In this issue, Raphael Mechoulam and his collaborators report novel data on cannabigerol derivatives able to reduce inflammation, pain and obesity, conditions where there is a huge unmet need of efficient drugs. Indeed, the interest in cannabigerol has been growing in the past few years and therapeutic expectations are rather high [9].

Allyn Howlett, the first Mechoulam Award recipient in 2000, John Huffman (also awarded in 2006) and Brian Thomas address the “spicy story” of cannabimimetic indoles, reviewing the discovery of aminoalkylindole analgesics, structure-activity relationship studies in search of their common pharmacophore, and their activity as cannabinoid receptor agonists [10].

George Kunos, awarded in 2005, and his colleagues describe novel findings on the effects of a peripherally restricted hybrid inhibitor of type 1 cannabinoid receptor (CB_1_) and inducible NO synthase (iNOS) on alcohol drinking behavior and alcohol-induced gut permeability. Of note, they analyze also the relative role of central versus peripheral CB_1_ receptors in alcohol drinking behavior, which may have major implications for drug discovery against alcohol dependence [11].

Vincenzo Di Marzo, awarded in 2007, reports new data on liver-expressed antimicrobial peptide-2 (LEAP-2) in the gut, showing that it is regulated by the endocannabinoidome-gut microbiome axis, an emerging and really hot topic in the field [12].

Ken Mackie, recipient of the Mechoulam award in 2008, examines with his colleagues the effects of several “minor” cannabinoids on neuronal function by using two model systems: cultured autaptic hippocampal neurons and dorsal root ganglion neurons. They show that two of these natural compounds (cannabidivarin and Δ^9^-tetrahydrocannabivarin) inhibit CB_1_ signaling, yet via distinct mechanisms [13].

Cecilia Hillard, who received the Mechoulam Award in 2011, reports that THC-induced catalepsy requires intact adenosine A_2_A receptor signaling to occur. She also shows that cannabidiol and its 4-fluoro derivative both can potentiate the cataleptic effect of THC, an effect that also requires A_2_A receptor signaling. Collectively, these data could be explained by cannabinoid inhibition of the equilibrative nucleotide transporter, which will raise adenosine concentrations thus resulting in activation of adenosine receptors, particularly A_2_A present in the striatum [14].

Beat Lutz, awarded in 2014, and colleagues describe subsynaptic distribution, lipid raft targeting and G protein-dependent signaling of CB_1_ in synaptosomes from the mouse hippocampus and frontal cortex. In summary, their results provide an updated view of the functional coupling of CB_1_ to G_αi/o_ proteins at excitatory and inhibitory terminals, and substantiate the utility of the CB_1_ rescue model in studying endocannabinoid physiology at the subcellular level [15]. Incidentally, CB_1_ location within lipid rafts remains an interesting subject of investigation after 15 years from its first discovery [16].

Mary Abood, who received the Mechoulam award in 2015, and her colleague review CB_1_ receptor signaling and biased signaling. The latter involves selective activation of a signaling transducer in detriment of another, mainly involving selective activation of G-protein or β-arrestin. However, biased signaling at the CB_1_ receptor is poorly understood due to the lack of strongly biased agonists. Mary also uses crystallographic structures of CB_1_ and proposed mechanisms of action of biased allosteric modulators to discuss a putative mechanism for CB_1_ activation and biased signaling [17].

Andreas Zimmer received the Mechoulam award in 2018, and with his colleagues reports new data on type 2 cannabinoid receptor (CB_2_) that is shown to alter social memory and microglial activity in an age-dependent manner. They demonstrate how physiological brain aging is characterized by gradual, substantial changes in cognitive ability, accompanied by chronic activation of the neural immune system, a relevant form of inflammation that is termed “inflammaging” [18].

Natsuo Ueda, 2020 Mechoulam awardee, and his coworkers describe the involvement of the γ-isoform of cytosolic phospholipase A_2_ (cPLA_2_) in the biosynthesis of bioactive *N*-acylethanolamines (NAEs) like *N*-arachidonoylethanolamine (anandamide), *N*-palmitoylethanolamine and *N*-oleoylethanolamine. In mammalian tissues NAEs are produced from glycerophospholipids via *N*-acyl-phosphatidylethanolamine (NAPE), and the ɛ isoform of cPLA_2_ functions as an *N*-acyltransferase to form this precursor. Since the cPLA_2_ family consists of six isoforms (α, β, γ, δ, ɛ, and ζ), the present study investigates a possible involvement of the isoforms other than ɛ in NAE biosynthesis. Presented results suggest that indeed cPLA2γ is involved in the biosynthesis of NAEs through its phospholipase A_1_/A_2_ and lysophospholipase activities [19].

Finally, Javier Fernandez-Ruiz, awarded in 2021, and his coworkers report a preclinical investigation on neuroprotective effects of the orphan G protein coupled receptor (GPR) 55 ligand VCE-006.1 in experimental models of Parkinson’s disease (PD) and amyotrophic lateral sclerosis (ALS). They conclude that targeting GPR55 may afford neuroprotection in PD, but not in ALS, thus stressing the differences in the development of cannabinoid-based therapies in neurodegenerative disorders [20].

This honorary issue of *Molecules* showcases contributions by half of the scientists who received the Mechoulam Award over the years. They are listed in Table 2 along with the awardees who unfortunately could not participate in this editorial project. I thank all colleagues for their valuable contributions to this volume, and I especially thank Professor Raphael Mechoulam for continuing to illuminate our field of research with his always inspiring new ideas.

## Figures and Tables

**Table 1:** Results of a PubMed search from 1964 (when THC was discovered) to 2021 with the entries “cannabinoids and disease” and “endocannabinoids and disease”. It should be recalled that the first endocannabinoid anandamide was discovered in 1992.

Time Range	Cannabinoids and Disease	Endocannabinoids and Disease
1964–1970	0	-
1971–1975	14	-
1976–1980	19	-
1981–1985	18	-
1986–1990	23	-
1991–1995	37	0
1996–2000	103	16
2001–2005	497	178
2006–2010	1305	665
2011–2015	1608	884
2016–2021	2924	1580

**Table 2:** Mechoulam Award recipients. Contributors to the present *Honorary Issue* are in italics.

Mechoulam Award Recipient	Year
*Allyn Howlett*	2000
Billy Martin	2001
Roger Pertwee	2002
Raj Razdan	2003
Murielle Rinaldi-Carmona and Francis Barth	2004
*George Kunos*	2005
*John Huffman* and Alex Makriyannis	2006
*Vincenzo Di Marzo*	2007
*Ken Mackie*	2008
Gerard Le Fur	2009
Patti Reggio	2010
*Cecilia Hillard*	2011
Ben Cravatt	2012
Aron Lichtman	2013
*Beat Lutz*	2014
*Mary Abood*	2015
*Mauro Maccarrone*	2016
Daniele Piomelli	2017
*Andreas Zimmer*	2018
Daniela Parolaro	2019
*Natsuo Ueda*	2020
*Javier Fernandez-Ruiz*	2021
